# Modelling Farmers’ Watershed Ecological Protection Behaviour with the Value-Belief-Norm Theory: A Case Study of the Wei River Basin

**DOI:** 10.3390/ijerph18095023

**Published:** 2021-05-10

**Authors:** Siyang Zhang, Minjuan Zhao, Qi Ni, Yu Cai

**Affiliations:** College of Economics and Management, Northwest A&F University, Yangling 712100, China; zsy18202955528@163.com (S.Z.); niqi@nwafu.edu.cn (Q.N.); caiyu1785@nwafu.edu.cn (Y.C.)

**Keywords:** extended value-belief-norm theory, watershed belonging, social capital, individual norm, watershed ecological protection behaviour, Wei River Basin

## Abstract

Encouraging farmers to protect their environment is of great significance in improving watershed ecological environments and promoting the sustainable development of the watershed economy. To explore the factors influencing farmers’ ecological protection behaviours in the river basin, we constructed a structural equation model to analyse the survey questionnaire responses of 719 farmers in the Wei River Basin, Shaanxi Province, China. The theoretical framework incorporated farmers’ watershed belonging and social capital into an extended value-belief-norm model. Robustness tests revealed that incorporating these variables was valid. Personality norms, watershed belonging, and social capital all had direct positive effects on farmers’ watershed ecological protection behaviour. Value orientation, environmental concern, consequences awareness, and responsibility attribution influenced the next variable in a causal chain and finally acted on watershed ecological protection behaviour indirectly through personality norms. Farmers’ watershed belonging and social capital positively impacted individual norm; through this, there was an indirect positive impact on their watershed ecological protection behaviour. Moreover, watershed belonging and social capital reinforced each other.

## 1. Introduction

Global environmental change is one of the hot topics in current international academic circles. The core issue of global environmental change is the increasingly worrying environment and development problems faced by human beings. At present, the research on global environmental change shows a general trend from focusing on the natural factors to emphasising the comprehensive effect of natural and human factors [1]. The current rapid development of urbanisation in China has entered the mid-term stage; but the fast urbanisation process has led to ever growing resource- and environment-protection-related problems. How to address the relationship between urbanisation and the ecological environment is of concern to academia and government decision-making departments as this represents a global strategic problem [2]. Human activities are the main driving force affecting ecological environment change and the key factor affecting the sustainable development of the ecological environment.

**Figure 1:**
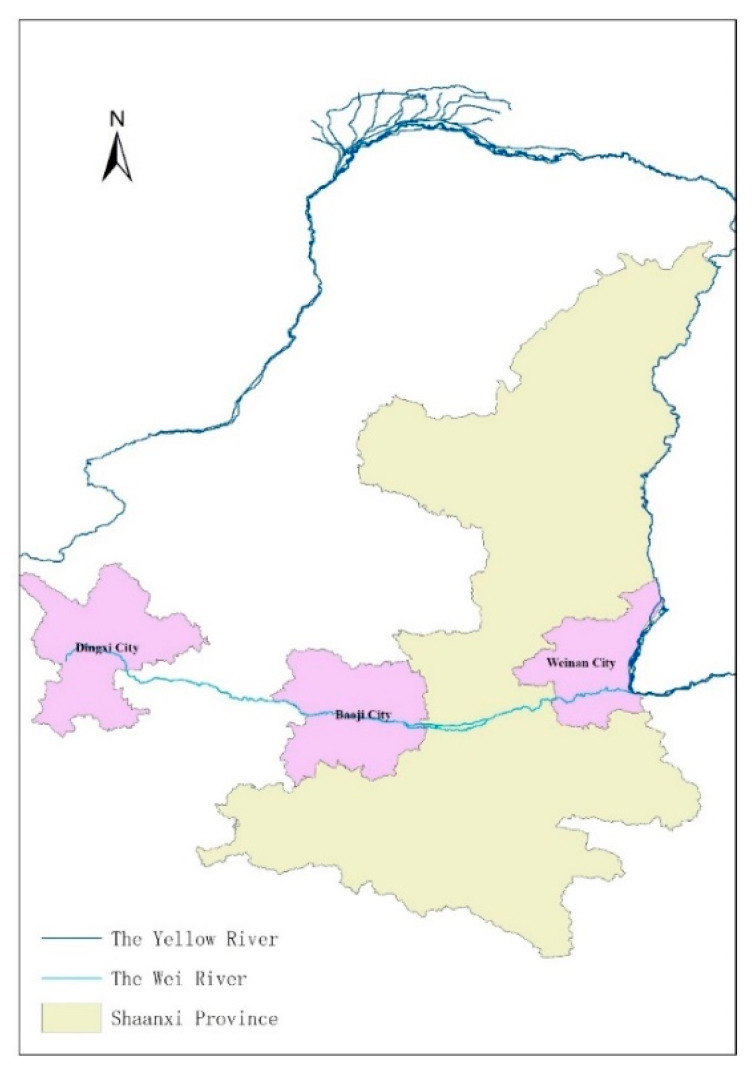
A map showing the geographical location of the Wei River.

The Yellow River Basin plays an important role in China’s economic and social development and ecological security. In August 2020, the Central Political Bureau of the Communist Party of China met to outline plans for the ecological protection and high-quality development of the Yellow River Basin (https://baijiahao.baidu.com/s?id=1676538485131713453&wfr=spider&for=pc (accessed on 31 August 2020)), noting its ecological and economic importance. Recognising its role in lifting people out of poverty, the Bureau aims to protect the basin’s ecology, promote high-quality development of the basin area, and thereby rejuvenate the Chinese nation and promote the national economy. As the largest tributary of the Yellow River, the Wei River plays an important role in promoting the ecological protection and high-quality development of the Yellow River Basin. The geographical location of the Wei River is shown in Figure 1.

Owing to urbanisation, industrial and agricultural production activities have recently begun to consume large volumes of water. Moreover, a large amount of water pollutant discharge has led to the Wei River Basin experiencing increasingly serious water shortages, water pollution, soil erosion, and other environmental problems in its ecologically sensitive areas [3]. Currently, the Wei River Basin is facing a serious shortage of water resources and the ecological environment of its watershed is deteriorating. Consequently, in November 2020, the management of the Wei River Basin in Shaanxi Province imposed regulations that prevent and control water pollution in the basin and improve its ecological environment. Governance of the Wei River Basin is an urgent and arduous task, which is of great significance for accelerating the economic and social development of the basin and surrounding regions and promoting their development. These policies improved the ecological environment of the Wei River Basin to a certain extent; but mostly focused on the strategic goals of the government and ignored the importance of farmers in protecting the Wei Basin’s ecological environment. As the mother river of the Shaanxi people, the Wei River is an important source of water for the production and life of the farmers along the river, and it plays an important role in the economic and social development of the province. As individuals with their own personal goals, farmers’ behaviours are influenced by their knowledge of watershed ecological protection practices and whether they have individual norms for implementing watershed ecological protection measures [4]. The active participation of farmers is an indispensable precondition for environmental protection [5]. Encouraging farmers to actively protect the ecological environment of river basins is an effective way to solve the problem of continuous deterioration of river basin ecosystems [6]. Therefore, to improve the ecological environment and promote the sustainable economic development of the Wei River Basin, it is essential to study the influencing factors and action paths of farmers’ watershed ecological protection behaviours.

The value-belief-norm (VBN) theory, which was proposed by Schwartz [7], is important in the field of social psychology and the study of prosocial behaviour. The theory considers three main variables: consequence awareness, responsibility attribution, and individual norm [7]. Stern extended the VBN theory by integrating the value theory and the new environmental paradigm theory, which connected value orientation, environmental concern, consequence awareness, responsibility attribution, and individual norm, and revealed their internal relationships [8]. Since then, the VBN theory has been widely applied to the study of pro-environmental behaviours, and it is regarded as the best theory for understanding all kinds of environmental protection behaviours [9]. In addition, social capital and sense of belonging also play a certain role in promoting the ecological protection behaviour of farmers in watersheds. There is an important, inextricable link between social capital and ecological environment protection [10]. The higher the level of social capital, the more the citizens will engage in behaviours that protect the environment [11]. Similarly, a sense of belonging encourages farmers to have the protection of the environment as their goal. The greater the farmers’ sense of belonging to a river basin, the stronger their sense of ownership [12]; the higher their degree of attachment to the basin, the higher will be the probability that they will actively protect the watershed ecology. Although existing literature has focused on the influence of sense of belonging on individual norms and behaviours, limited research has explored the role that sense of belonging plays in watershed ecological protection behaviour—studies have generally ignored the role of emotional factors, such as sense of belonging. Concurrently, rural society is fortunate as farmers’ social networks tend to be strong: the activation of farmers’ individual norms concerning their ecological protection behaviours in the basin is influenced by the views and practical actions of their relatives, friends, and neighbours [13].

The VBN theory has been continuously confirmed and expanded since it was first proposed. It has excellent power to explain individual environmental protection behaviours [13]. Sense of belonging and social capital also have an important impact on individual norms and behaviours. However, limited studies have used the VBN theory to study the ecological protection behaviours of farmers in river basins, nor has the theory incorporated the sense of belonging and social capital into theoretical models that explore the occurrence of ecological protection behaviours in river basins. Farmers’ values and motivations have an important impact on the environment and arise from their intrinsic conscience and sense of responsibility. Together, they activate farmers’ awareness and concern, and the standards to which they hold themselves [7]. Concurrently, individual behaviour norms and watershed ecological protection are affected by farmers’ sense of belonging and social capital. Therefore, this study, based on a survey of 719 farmers in the Wei River Basin in Shaanxi Province, used the extended VBN theory model, and introduced sense of belonging and social capital into the theoretical framework. Our aims were to study farmers’ watershed ecological protection behaviour, find ways of encouraging farmers to participate in the development of a river basin ecological protection policy on a scientifically robust basis, and make a valuable contribution to the existing literature.

## 2. Theoretical Analysis and Research Hypothesis

The VBN framework considers five main variables: value orientation, environmental concern, consequence awareness, responsibility attribution, and individual norm. These five variables link to form an unbreakable causal chain. Each variable in the chain is directly related to the next variable and may also be related to variables further along the chain [7].

**Figure 2:**
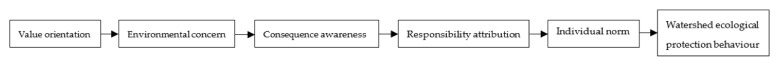
Initial value-belief-norm theory model.

According to the VBN theory, farmers’ ecological protection behaviour is activated by individuals’ characteristics; specifically, the characteristics that motivate farmers not to engage in antisocial behaviour and to avoid damage to others (i.e., awareness of consequence). Farmers experience adverse consequences for not behaving in prosocial ways. The farmers’ awareness of consequences and attribution of responsibility are influenced by their personal values and their orientation towards environmental care [7]. Specifically, the VBN theory, based on the theory of values, is a refined personal value system, and the environment is closely related to the three kids of value orientation. The main body of behaviour is formed under the effect of different value orientations. Specific environmental concerns will cause different ways of thinking about environmental issues, trigger appropriate senses of concern and responsibility, and stimulate different aspects of an individual’s character, leading to positive or negative environmental protection behaviour [14]. The initial VBN theoretical model used in this study to examine the ecological protection behaviours of farmers in the watershed is shown in Figure 2.

### 2.1. The Influence of Value Orientation, Environmental Concern, Consequence Awareness, and Responsibility Attribution on Individual Norm

Value orientation refers to individuals’ [15] ecological world outlook; that is, their ecological values. Value orientation is the basis of research on public environmental protection behaviour, the first variable in the causal chain of the VBN theoretical model, and the most basic research variable. Stern and colleagues [16] believe that different value orientations will directly affect the environmental protection behaviours of individuals. Value orientation can be divided into three categories: (1) self-interested value orientation: the individual tries to maximise individual interests and often pursues the maximisation of his or her own economic interests, reflecting the characteristics of “economic man”; (2) altruistic value orientation: while engaging in environmental protection behaviours, the individual will pay attention to the welfare of other people and strive to maximise their interests; and (3) biosphere value orientation: while engaging in environmental protection behaviours, the individual will not only consider the welfare of other people, but also pay attention to the interests of non-human species [17]. However, in general, value orientation does not have a strong direct impact on environmental protection behaviour, and the relationship between the two is usually mediated by environmental concern and individual norm [18].

Environmental concern refers to individuals’ concern for the surrounding environment, which is closely related to their personal norms and specific behaviours [19]. The new environmental paradigm (NEP), as a representative theory of environmental concern, has been studied extensively. It was first proposed by the American environmental sociologists Catton and Dunlap [20]. The NEP attaches great importance to the impact and constraints of environmental factors on human society and posits that human behaviours have caused sustained adverse impacts on the fragile ecological environment. Dunlap and colleagues believe that the more people accept this NEP, the brighter will be the prospects for environmental improvement [21]. Similar to value orientation, the relationship between environmental concern and ecological protection behaviour is usually not strong [22], and the relationship between the two is also mediated by consequence awareness and responsibility attribution.

Consequence awareness refers to individuals’ awareness of the adverse consequences caused by their failure to have a prosocial approach to other people or objects, while responsibility attribution refers to individuals’ belief that they are responsible for the adverse consequences caused by that failure [7]. Many empirical studies have shown that when the levels of consequence awareness and responsibility attribution are high, individual norms are more likely to guide the corresponding behaviour [23,24].

Individual norm is a concept first put forward by Schwartz, who defined it as the internalising of social norms and a sense of moral obligation, the violation of which can produce feelings of guilt, self-denial, or loss of self-esteem; whereas, abiding by individual specification will generate a sense of pride and self-esteem [25]. Individual norm is the most direct and closest variable to environmental behaviour in the VBN theory [7].

According to the VBN theory, farmers’ value orientation towards watershed ecological environment protection determines their level of environmental concern. Farmers with more concern for the environment are more likely to be aware of the adverse consequences of non-environmental protection behaviours for watershed ecology, thus making environmental concern an intermediate variable between value orientation and consequence awareness. Individual behaviour norms have a positive relationship with farmers’ basin ecological protection behaviour only when the farmers admit that there needs to be attribution of responsibility for their actions [26]. To activate farmers’ individual specifications, they must realise that if they do not implement river basin ecological protection behaviour, they will bring about negative results, and further that they have a responsibility to protect the basin’s ecological environment. Therefore, this study proposes the following hypotheses (H):

**Hypothesis** **1** **(H1).** *Value orientation will have a positive impact on environmental concern.*

**Hypothesis** **2** **(H2).** *Environmental concern will have a positive effect on consequence awareness.*

**Hypothesis** **3** **(H3).** *Consequence awareness will have a positive influence on responsibility attribution.*

**Hypothesis** **4** **(H4).** *Responsibility attribution will have a positive impact on individual norm.*

### 2.2. Impacts of Individual Norm, Social Capital, and Watershed Belonging on Farmers’ Watershed Ecological Protection Behaviour

The individual norm is a moral obligation based on personal values and a sense of responsibility “to do the right thing” [27]. If farmers regard watershed ecological protection as their moral obligation, they will be inclined to protect watershed ecology. According to Onwezen, observing individual norm will make individuals generate feedback about their own emotions, which will strengthen their altruistic behaviours [28].

Sense of belonging refers to an individual’s identification, and the strength of their relationship, with an object or phenomenon [12]. In this study, the sense of belonging to a basin was defined as the emotional connection between farmers and the people and environment in the basin that they inhabit. Only when farmers have feelings of identification, affection, and attachment to a basin will they actively pay attention to its construction and development and consciously protect its ecological environment. The sense of belonging will encourage farmers to establish collective behavioural goals, which is conducive to promoting their sense of mutual understanding and community and the establishment of individual norm [29]. Similarly, farmers with a strong sense of belonging to a watershed are more likely to regard the individual norm of protecting the watershed ecology as their personal goal, have a positive attitude towards the watershed ecology, and thus, spontaneously protect the watershed ecology. In addition, farmers with a sense of belonging are more willing to bear more responsibilities and obligations to seek long-term collective development [30]. Therefore, residents with a stronger sense of belonging to the basin are more inclined to protect the ecological environment of the basin in their own long-term interest.

The concept of social capital was first formally proposed by Bourdieu, who believed that it derives from the emotional relationship or resource exchange generated by people’s communication processes and is a relationship network that helps participants obtain real or potential social resources [31]. Putnam further developed the concept of social capital, seeing it as a network, trust, and norm that can improve social efficiency by promoting cooperation [32]. Watershed ecological protection is a form of public resource management and has the attribute of a common good that requires the collective participation of farmers to survive and thrive; social capital is key to fostering collective action [32,33]. Through a case analysis, Ostrom found that social capital, such as social networks, social trust, and behavioural norms formed through people’s long-term communication processes, plays an important role in reducing “free rider” behaviour [34], thus contributing to the establishment of prosocial personal norms. Concurrently, Hengtong and colleagues believed that social capital, as an internal incentive mechanism, has a strong positive influence on farmers’ participation in watershed ecological protection activities [35]. Yuxing and colleagues proposed that an improvement of social capital is conducive to promoting herders’ participation in grassland community governance [36]. It is evident that social capital is an indispensable part of environmental protection [37] and an important force driving people to participate in environmental protection [38]. Consequently, we proposed the following:

**Hypothesis** **5** **(H5).** *Individual norm will have a positive impact on watershed ecological protection behaviour.*

**Hypothesis** **6** **(H6).** *Watershed belonging will have a positive influence on individual norm.*

**Hypothesis** **7** **(H7).** *Watershed belonging will have a positive influence on watershed ecological protection behaviour.*

**Hypothesis** **8** **(H8).** *Social capital will have a positive impact on individual norm.*

**Hypothesis** **9** **(H9).** *Social capital will have a positive influence on watershed ecological protection behaviour.*

### 2.3. The Influence of Watershed Belonging, and Social Capital

Watershed belonging comprises farmers’ affection for, attachment to [39], psychological identification with, and dependence on the regional environment and the local population [40]. Farmers’ need for a sense of belonging is an endogenous driving force underlying the formation of social capital [41]. Farmers’ watershed belonging encourages them to establish good social relationships [42] and form a harmonious community. In particular, farmers with a stronger sense of belonging are more willing to invest time and energy to sustain themselves in the community in the river region; they have better social relationships and show more prosocial characteristics, such as trust, willingness to cooperate with others, and more frequent attendance at social gatherings [38]. Additionally, we should consider the strong heterogeneity of farmers. To make this diverse group of farmers establish a strong sense of belonging to the basin, the highest priority is to foster close and lasting relationships between them: to cultivate social capital [43]. Watershed belonging to a watershed is based on human feelings and familiarity. Social capital can generate emotional tendencies, such as trust, understanding, and empathy among farmers, thus promoting the formation and development of one’s sense of belonging. Social capital connects farmers in the basin area in the form of social bonds, which enhance the relationships between farmers. Specifically, when farmers’ social capital is enhanced, their communication and interaction with the people around them will increase, their mutual trust will be strengthened, and their relationships will be closer [42]. This will strengthen farmers’ positive feelings towards the people and the environment in the basin area, and further enhance their sense of belonging to the basin. Consequently, we proposed the following:

**Hypothesis** **10** **(H10).** *Watershed belonging will have a positive influence on social capital.*

**Hypothesis** **11** **(H11).** *Social capital will have a positive influence on watershed belonging.*

### 2.4. Impacts of Value Orientation, Environmental Concern, Consequence Awareness and Responsibility Attribution to the Watershed on Farmers’ Watershed Ecological Protection Behaviour

According to the variable path of the initial value-belief-norm theory, value orientation [15], environmental concern [19], outcome awareness, and responsibility attribution [20,21] have no direct influence on farmers’ ecological protection behaviour in river basins. However, some scholars have proposed that the above four variables have significant direct influence on farmers’ ecological protection behaviour in river basins. Heesup [44] believed that value orientation plays an important role in promoting environmental protection. Tam and Chan [45] found that environmental concern can significantly positively affect human environmental protection behaviour. Liobikienė and Juknys [46] believed that people with a strong sense of outcome will enhance their willingness to protect the environment and then have environment-friendly behaviours. Fielding and Head [47] proposed that people with stronger responsibility attribution were more inclined to protect the environment, and responsibility attribution was an important factor affecting environmental protection behaviour. Therefore, in order to comprehensively investigate the mechanism of farmers’ watershed ecological protection behaviour, this paper will also study this issue. Consequently, we proposed the following:

**Hypothesis** **12** **(H12).** *Value orientation will have a positive impact on watershed ecological protection behaviour.*

**Hypothesis** **13** **(H13).** *Environmental concern will have a positive impact on watershed ecological protection behaviour.*

**Hypothesis** **14** **(H14).** *Consequence awareness will have a positive impact on watershed ecological protection behaviour.*

**Hypothesis** **15** **(H15).** *Responsibility attribution will have a positive impact on watershed ecological protection behaviour.*

**Figure 3:**
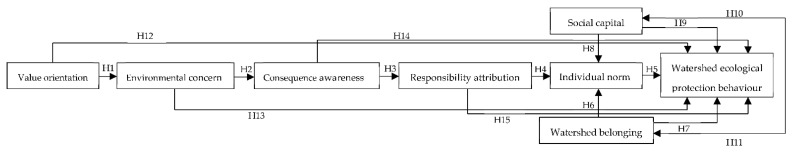
Extended value-belief-norm theory model.

Combining these hypotheses with the theories described above, the model constructed in this study is shown in Figure 3.

## 3. Methods and Materials

### 3.1. Data Sources

With a total length of 818 km and a basin area of 134,800 km^2^, the Wei River Basin has an important impact on regional economic development and strategy for large-scale development of Western China. The data used in this study were obtained from a field household survey performed by the research team in the Wei River Basin, Shaanxi Province, in October 2018. We conducted face-to-face interviews with respondents and in accordance with relevant ethics requirements. Baoji City and Weinan City are in the middle and lower reaches of the Wei River, respectively. Shaanxi Province has provided certain ecological compensation funds to the upper reaches of the Wei River for pollution control, ecological protection of water source and water quality monitoring, etc. Therefore, farmers in the middle and lower reaches need to respond to the call of the government and actively protect the ecological environment of the river basin. Baoji City and Weinan City are shown in Figure 1. In this paper, stratified sampling and random sampling were used to select two cities and four counties (districts). A total of 750 questionnaires were distributed. After eliminating invalid responses, 719 valid questionnaires were finally obtained (response rate = 95.87%; Table 1).

As seen in Table 2, the interviewed farmers had the following characteristics: there were slightly more male farmers (53.96% of the total sample size) than female ones and the respondents were generally older (58.42% were older than 46 years). The interviewed farmers were not highly educated; those with only a junior middle school education accounted for 34.21% of the total. Of the respondents, 96.11% had never been engaged in a career related to environmental protection, and most did not believe that they had experienced the ecological value of “harmonious coexistence and coordinated development between man and nature” advocated by strategy of ecological civilisation. The annual farmer income of the interviewed farmers was generally low, with 54.80% of the farmers having an annual farmer income below 60,000 Yuan (7698 Euros).

### 3.2. Measurement of Variables

Based on the VBN theory, this study selected value orientation, environmental concern, consequence awareness, responsibility attribution, individual norm, watershed belonging, social capital, and watershed ecological protection behaviour as the latent variables of the structural equation model (SEM). Among these, value orientation encompasses the observed variables arising from the three aspects of self-interest, altruism, and beneficence towards the biosphere; the other specific measurement items are shown in Table 3. The measurement items for the variables were developed specifically for this study and they were pre-tested and deemed effective. A Likert scale was used to measure the items, with 1 indicating complete disagreement and 5 indicating complete agreement; thus, the larger the score, the greater the agreement. In addition, considering that sex, age, education level, family income levels, professional skills, and other factors affect farmers’ ecological protection behaviour [35,48,49], this study measures these variables in relation to environmental protection as they may have an influence independent of the controlled variables.

### 3.3. Model Construction

All the latent variables in the model contain multiple observed variables; thus, to verify the complex causal relationships between variables, we adopted an SEM for empirical testing. Compared to other methods, SEM has the advantage of addressing the relationships between dominant indicators and latent variables, test the fit between data and theoretical framework, and include error terms in the model [50]. Therefore, it is an appropriate method to test the model in this study. The specific SEM form was as follows:

*Y* = *λ_y_η* + *ε* (1)

*X* = *λ_x_ξ* + *σ* (2)

*η* = *Bη* + *Γξ* + *ζ* (3)

where Equations (1) and (2) are measurement equations that reflect the consistent relationship between latent and observed variables, *Y* is the observed variable vector of endogenous latent variable, *X* is the observed variable vector of the exogenous latent variable, *λ_y_* is the factor loading matrix of the endogenous latent variable and its observed variable, and *λ_x_* is the factor loading matrix of the exogenous latent variable and its observed variable. Equation (3) is the structural equation that links the endogenous with the exogenous latent variables. In Equation (3), *η* is the endogenous latent variable; *ξ* is the exogenous latent variable; *B* is the endogenous latent variable coefficient matrix; *Γ* is the exogenous variable coefficient matrix; and *ε*, *σ* and *ζ* are the error terms.

## 4. Results

### 4.1. Results of Reliability and Validity Testing

To test and analyse the reliability and validity of the questionnaire, this paper used SPSS 23.0 (IBM, Armonk, NY, USA) to test and analyze them, and the test results are shown in Table 4. The Cronbach’s alpha coefficient for the complete table of variables was 0.732. For protection behaviour, value orientation, environmental concern, consequence awareness, and individual norm, the Cronbach’s alpha coefficient values all exceeded the ideal level of 0.7 [51]. For responsibility attribution, watershed belonging, and social capital, the Cronbach’s alpha values were more than 0.6; thus, they were deemed acceptable [51]. The questionnaire was thus considered reliable. The Kaiser–Meyer–Olkin (KMO) test for sampling adequacy, the Bartlett sphericity test, and factor loading coefficients were also used to test the validity of the questionnaire. For all the variables, the KMO was 0.692; for the watershed belonging variable, the KMO value was 0.500; and for the other variables, the KMO values exceeded 0.6 [51]. The Bartlett sphericity test values were significant (*p* < 0.001). In addition, the factor loadings of each observed variable coefficient all exceeded 0.6, indicating that the questionnaire had a sound structure validity, and was sufficiently valid to conduct a factor analysis [51]. In sum, the questionnaire was deemed valid.

### 4.2. Results of Model Overall Fitness Test

Model adaptation degree is an important basis for testing whether the extension of the basic VBN model is scientific and robust [52]. This study used AMOS 23.0 to test the initial model and the extended model adaptation. The test results are shown in Table 5. Compared to those in the initial model, each index value of the extended model was an improvement. Therefore, it is reasonable and scientific to include watershed belonging and social capital in the VBN theoretical model framework to study farmers’ watershed ecological protection behaviours.

### 4.3. Model Estimation Results

AMOS 23.0 was used to estimate the paths between the latent variables of the SEM; the calculated model estimation results are shown in Table 6.

## 5. Discussion

The results of the standardised path coefficient of the influence of value orientation on the environmental concern path was 0.216 (*p* < 0.05). Additionally, the influence of environmental concern on consequence awareness had a path coefficient of 0.314; the influence of consequence awareness on responsibility attribution had a standardised path coefficient of 0.284; responsibility attribution on the individual norm had a path coefficient of 0.448; all these three pathways were significant (*p* < 0.01). The results show that farmers, under the influence of value orientation, developed environmental concern, and specific environmental concerns induced ecological thinking among them; thus, the more environmental concern farmers have, the more likely they will be aware of the adverse consequences of not having positive watershed ecological environmental behaviour, prompting in them the consciousness to pursue ecological results. In short, the more aware the farmers are of the adverse consequences of not implementing ecological protection in the river basin, the stronger their sense of responsibility to protect the river basin will be [24]. Because they know the severity of the adverse consequences, they will take environmental protection as their responsibility and try their best to protect the river basin’s ecological environment. In the end, the farmers’ attribution of responsibility stimulates their individual norms and urges them to adopt ecological protection behaviours consistent with their individual norms. The above four paths belong to the initial value-belief-norm theoretical model, which has been verified by many scholars and has led to a consistent conclusion. Therefore, H1, H2, H3, and H4 were supported.

The standardised path coefficient of the influence of individual norm on watershed ecological protection behaviour was 0.874 (*p* < 0.01). This shows that individual norm had a positive effect on the ecological protection behaviour of farmers in the basin, and that the improvement of individual norm helps promote the protection of the ecological environment of the basin. The standardised path coefficient of the influence of watershed belonging on individual norm was 0.160 (*p* < 0.01). This indicates that strengthening the farmers’ sense of belonging in the basin helps them improve their individual norms. Concurrently, the standardised path coefficient of the influence of watershed belonging on watershed ecological protection behaviour was 0.872 (*p* < 0.01). This indicates that the sense of belonging of farmers in the basin had a direct positive effect on their ecological protection behaviour; and that the stronger their sense of belonging, the more farmers will actively protect the ecological environment of the basin. Watershed belonging is the emotional connection between the farmers, people, and the environment in the basin where they live. Watershed belonging promotes a sense of community among farmers and promotes the establishment of individual norm [27]. At the same time, farmers with watershed belonging are more willing to undertake more responsibilities and obligations in order to seek long-term collective development [28]. Therefore, the residents with a stronger watershed belonging are more inclined to take the initiative to implement environmentally friendly behaviours in the basin and actively protect the ecological environment in the basin. In addition, the standardised path coefficient of the influence of social capital on individual norm was 0.209 (*p* < 0.01), which indicates that farmers’ social capital also plays a key role in promoting the improvement of individual norm. The standardised path coefficient of the influence of social capital on watershed ecological protection behaviour was 0.788 (*p* < 0.01). This indicates that the social capital of farmers had a direct positive effect on their watershed ecological protection behaviour; that is, farmers with more social capital were more inclined to protect the watershed ecological environment. River basin ecological protection belongs forms part of public resource management, and social capital is the key to cracking the collective action dilemma [30,31], because social capital promotes cooperation between the farmers and improves interpersonal trust and specification [30] which helps set up correct individual specifications. At the same time, as an internal incentive mechanism, social capital can also directly promote farmers’ participation in ecological protection behaviour [33,34]. Social capital can promote farmers’ ecological environmental protection behaviour in the river basin, and farmers with more social capital tend to protect the ecological environment in the river basin. Therefore, scholars generally believe that watershed belonging and social capital are indispensable components of people’s participation in environmental protection [35]. Thus, H5, H6, H7, H8, and H9 were verified.

The standardised path coefficient of the influence of social capital on watershed belonging was 0.469 (*p* < 0.01). In a typical rural society of China, the more frequent the exchanges between farmers and their relatives and neighbours, the deeper will be their feelings towards the basin area, and the closer will be their ties with the group members in the basin area. Therefore, social capital plays a positive role in promoting watershed belonging. From another point of view, in order to cause different farmers to develop a high sense of belonging in the same basin, the first step is to create a close and lasting interactive relationship between them, which is a method for cultivating social capital [41]. Social capital can generate trust, understanding and empathy among farmers and promote the formation and development of a sense of belonging in the basin. To be specific, when farmers’ social capital increases, their communication and interaction with the people around them will increase, their mutual trust will be strengthened, their feelings will be deepened, and the relationship between them will be closer [40], which will strengthen farmers’ feelings for the people and environment in the basin and enhance their sense of belonging to the basin. The standardised path coefficient of the influence of watershed belonging on social was 0.694 (*p* < 0.01). Farmers with a strong sense of belonging to the basin were more willing to spend time and energy communicating with people around them, which deepens their trust in, and intimacy with, the people. Therefore, watershed belonging also promoted the improvement of farmers’ social capital. Here, watershed belonging means that farmers and other residents residing in the same environment will produce a mutual psychological identity and dependence on the living environment [38], psychological demand of farmers to seek for the formation of social capital provides the endogenous power [39], strong sense of belonging in farmers, more willingness to invest time and effort to communicate with people around, to side person’s trust and will also be increased as the close degree. Having watershed belonging can easily form a community with harmonious relationship among farmers in the basin, so a sense of belonging in the basin can promote the improvement of farmers’ social capital and encourage them to show more prosocial characteristics, such as more trust in others, more willingness to cooperate with others, and more frequent participation in social gatherings [36]. Therefore, the positive relationship between social capital and watershed belonging has also been recognized in academic circles. Thus, H10 and H11 were verified.

Value orientation, environmental concern, consequence awareness and responsibility attribution have no significant effect on the standardization path of farmers’ watershed ecological protection behaviour, which proves that all four variables influence the next variable successively through the causal chain, and finally have an indirect impact on the watershed ecological protection behaviour through individual norm, rather than a direct impact. In view of the differences in the conclusions of previous studies by different scholars, we believe that the different situations in different parts of the world and the factors that affect people’s environmental protection behaviours are different, other variables selected by various scholars are different, and the variables may influence each other, thus resulting in such path differences. In this study, there is no significant direct influence between these last variables, thus overturning H12, H13, H14, and H15 mentioned above. This also verifies the applicability of value-belief-norm theory in this paper.

According to the theoretical model described above, farmers’ watershed belonging and social capital can affect their watershed ecological protection behaviour, not only directly, but also indirectly by influencing their individual norms. To this end, we further tested and analysed the mediating effects of individual norms on the relationships between watershed belonging, social capital, and watershed ecological protection behaviours (Table 7). The results show that the indirect effects of farmers’ watershed belonging and social capital on watershed ecological protection behaviours were 0.140 and 0.183, respectively. In addition, the effect of individual norm on watershed ecological protection behaviour was partially mediating, while the indirect effect of individual norm on watershed ecological protection behaviour was smaller than the direct effect, indicating that watershed belonging and social capital mainly promotes farmers’ watershed ecological protection behaviour through their direct effect.

## 6. Conclusions

### 6.1. Research Conclusions

This study used an extended VBN theoretical framework to analyse the ecological protection behaviour of farmers in the Wei River Basin in China’s Shaanxi Province. We used data from a survey of 719 farmers, adopting an SEM, and drew the following conclusions:

1. The model based on the VBN theory was successfully applied to farmers’ behaviour in the study of river basin ecological protection. By introducing the notions of watershed belonging, social capital provides a new, rational, and scientific basis to explain farmers’ river basin ecological protection behaviour. Not only did this enrich the VBN theory for this study, but it also makes the theory closer to the reality of China.
2. Individual norm, watershed belonging, and social capital had direct, positive impacts on watershed ecological protection behaviours. Of these factors, the direct effect of individual norm was the strongest, followed by watershed belonging. The effect of social capital was the weakest.
3. Value orientation, environmental concern, consequence awareness, responsibility attribution, and individual norm constituted the VBN causal chain, with each variable, in turn, positively influencing the next variable and, finally, indirectly influencing watershed ecological protection behaviour through individual norm. Concurrently, individual norm was also positively influenced by sense of belonging and social capital. Through their influence on individual norms, they also have an indirect positive influence on the ecological protection behaviour of the basin.
4. There is a mutually reinforcing relationship between watershed belonging and social capital. Sense of belonging to the basin was conducive to the formation and maintenance of farmers’ social capital, and social capital was conducive to the enhancement of farmers’ sense of belonging to the basin.

### 6.2. Implications, Limitations, and Future Research

Based on these conclusions, the following suggestions to promote farmers’ ecological protection behaviour in the Wei River Basin are proposed:

1. Based on the VBN theory, this paper includes the emotional factor: watershed belonging, and adds the variable of social capital in combination with the actual situation in rural China, which enriches the VBN theory and makes the theory closer to the reality of China.
2. To foster farmers’ ecological values, they should be educated about the environment and encouraged to identify with the watershed, as well as adopt positive watershed ecological protection behaviours. Farmers should be made aware of the consequences of the destruction of the river basin ecological environment. Additionally, their awareness of liability should be promoted. These measures will promote norms favouring the protection of the Wei River Basin ecology.
3. Publicity campaigns should be actively carried out to enhance farmers’ watershed belonging. In addition to more publicity about the need to protect the watershed ecological environment through publicity boards, radio, television, and other media, education and publicity on watershed ecological protection behaviours directly related to the farmers’ well-being should be availed in various forms to deepen their understanding of the importance of watershed ecological protection in their lives and farm productivity. This study emphasised the importance of farmers’ ownership status, their affection for and attachment to the watershed, and the psychological and emotional factors that can be guided in ways that encourage watershed ecological protection.
4. Recognising the leading role that social organisations, party members, and village cadres can play a vital role in promoting the social capital of farmers, raising their awareness of the need to protect the environment, offering practical advice, generating their enthusiasm to protect the river basin, and fostering prosocial environmental behaviours.

Owing to the limitation of time and funds, this study combined stratified sampling and simple random sampling, and only 719 farmers in the middle and lower reaches of Wei River were examined. The data used cannot reflect the overall situation of ecological protection in the watersheds of China or even the world. Farmers in regions with different economic levels may display diverse behaviours. Farmers in other areas should be examined in the future. When comparing different basins, we should pay attention to the differences in geographical location, economic development mode, and national policies. In addition, the influence of the protection behaviours of farmers on each other is a complex issue that requires further in-depth investigation.

## Figures and Tables

**Table 1:** Questionnaire distribution.

Province	Cities	Counties (Districts)	Number Distributed	Number of Valid Responses	Response Rate (%)
Shaanxi	Baoji	Chencang	188	181	96.28
		Mei	194	187	96.39
	Weinan	Linwei	185	176	95.14
		Tongguan	183	175	95.63
Total			750	719	95.87

**Table 2:** Basic characteristics of the interviewed farmers.

Basic Information	Classification	Number of Persons	Proportion (%)
Sex	Male	388	53.96
	Female	331	46.04
Age (years)	≤25	42	5.84
	26–35	142	19.75
	36–45	115	15.99
	46–55	153	21.28
	55–65	156	21.70
	>65	111	15.44
Education	Never attended any school	29	4.03
	Primary school	102	14.19
	Junior high school	246	34.21
	High school or technical secondary school	151	21.00
	Junior college	100	13.91
	Bachelor’s degree or above	91	12.66
Engaged in a career related to environmental protection	Yes	28	3.89
	No	691	96.11
Annual farmer income (10,000 Yuan, 1277 Euros)	≤3	166	23.09
	3–6	228	31.71
	6–9	154	21.42
	9–12	88	12.24
	>12	83	11.54

**Table 3:** Measurement items and descriptive statistics of variables.

Variable Category	Measurement Items	Code	Mean	SD
Watershed ecological protection behaviour	If someone destroys the ecological environment of the Wei River Basin, I will not hesitate to complain or report this to the relevant administrative departments	PB1	3.277	1.049
	If someone destroys the ecological environment of the Wei River Basin, I will question whoever s/he is	PB2	3.092	0.901
	If ecological protection and pollution control are performed in the Wei River Basin upstream, the ecological environment of this region will be improved, and I am willing to pay certain fees to the upstream region for this	PB3	3.057	0.438
Value orientation	Pollution control and environmental protection can increase the amount of water available in my daily life	VO1	3.708	1.027
	Pollution control and environmental protection can increase the amount of water available for agricultural irrigation in the surrounding areas	VO2	3.644	1.042
	Pollution control and environmental protection can increase the variety of rare aquatic life in the river basin	VO3	3.631	1.147
Environmental concern	In the past five years, the ecological environment of the Wei River Basin in my region has become very good	EC1	3.769	0.809
	In the past five years, the water quality of the Wei River Basin in my area has become very good	EC2	3.139	0.920
	In the past five years, the amount of water in the Wei River Basin in my area has increased	EC3	2.704	0.870
	In the past five years, the number of fish species in the main stream of the Wei River Basin in my area has increased	EC4	2.677	0.884
Consequence awareness	If pollution control measures are not taken, it will lead to pollution of livestock and poultry breeding, and dirty urban water bodies with a foul smell	CA1	3.623	1.088
	If we do not increase investment in environmental protection, we will fail change to drought-tolerant economic crops, reduce agricultural irrigation water consumption, and thus will fail better develop the environmental protection industry	CA2	3.670	0.973
	If there is no pollution control and environmental protection work upstream, the water quality of the incoming section will become worse	CA3	3.997	1.011
	If pollution control and environmental protection are not performed upstream, the area for eco-tourism and recreation will be reduced	CA4	4.028	0.939
Responsibility attribution	I have the responsibility to improve the ecological environment of the Wei River Basin and prevent water pollution	RA1	4.433	0.714
	I have the responsibility to pay a certain fee to the protectors of the ecological environment in the Wei River Basin (the groups who have made certain sacrifices to protect the ecological environment)	RA2	3.262	0.723
	It is my duty to understand the term “watershed ecological compensation”	RA3	3.572	0.818
Individual norm	I should contribute to the ecological environment improvement of the Wei River Basin	IN1	3.104	1.032
	I should improve the ecological environment of the Wei River Basin by changing my daily production activities and lifestyle	IN2	3.604	0.949
	I should make some changes to improve the ecological environment of the Wei River Basin (such as saving water, reducing the use of fertilisers and pesticides in agricultural production)	IN3	3.727	0.902
Watershed belonging	My family lived in the Wei River Basin for a long time	WB1	3.672	1.003
	I have a strong sense of belonging to the Wei River Basin	WB2	4.134	0.859
Social capital	I have great trust in my friends and neighbours	SC1	2.210	1.159
	I have frequent contact with friends and neighbours	SC2	2.320	1.197
	I often take part in group activities in the village	SC3	2.633	1.227
	I often give suggestions or opinions when making public affairs decisions in the village	SC4	2.298	1.219
Control variable	Sex (male = 1, female = 0)	SEX	0.540	0.499
	Age (years)	AGE	48.775	15.216
	Education (Never attended any school = 1, Primary school = 2, Junior high school = 3, High school or technical secondary school = 4, Junior college = 5, Bachelor’s degree and above = 6)	EDU	3.644	1.343
	Annual farmer income (10,000 Yuan)	INC	6.901	5.805
	Engaged in a career related to environmental protection (yes = 1, no = 0)	ENV	0.039	0.194

Note: SD = standard deviation.

**Table 4:** Results of reliability and validity tests.

Latent Variable	Observational Variable	Factor Loading	Cronbach’s α	KMO	Bartlett Sphericity Test	Significance Level
Watershed ecological protection behaviour	PB1	0.846	0.752	0.682	454.452	<0.001
	PB2	0.815				
	PB3	0.714				
Value orientation	VO1	0.624	0.738	0.661	340.180	<0.001
	VO_2_	0.716				
	VO3	0.725				
Environmental concern	EC1	0.713	0.702	0.670	319.051	<0.001
	EC2	0.742				
	EC3	0.609				
	EC4	0.705				
Consequence awareness	CA1	0.707	0.749	0.701	493.217	<0.001
	CA2	0.711				
	CA3	0.664				
Responsibility attribution	RA1	0.755	0.697	0.681	213.379	<0.001
	RA2	0.636				
	RA3	0.741				
Individual norm	IN1	0.802	0.807	0.785	771.288	<0.001
	IN2	0.893				
	IN3	0.860				
Watershed belonging	WB1	0.768	0.631	0.500	183.801	<0.001
	WB2	0.768				
Social capital	SC1	0.745	0.633	0.639	730.555	<0.001
	SC2	0.726				
	SC3	0.660				
	SC4	0.672				
Total			0.732	0.692	3702.403	<0.001

**Table 5:** Result of model overall fitness test.

Index	Initial Model Index Values	Extended Model Index Values	Fitness Requirement	Fitness Evaluation
*χ^2^/* *df*	2.888	1.813	1 < 1.813 < 3	Ideal
NFI	0.864	0.927	0.927 > 0.9	Ideal
RFI	0.852	0.910	0.910 > 0.9	Ideal
IFI	0.907	0.950	0.950 > 0.9	Ideal
TLI	0.884	0.937	0.937 > 0.9	Ideal
CFI	0.906	0.950	0.950 > 0.9	Ideal
PNFI	0.700	0.712	0.712 > 0.5	Ideal
PCFI	0.734	0.754	0.754 > 0.5	Ideal
RMSEA	0.051	0.034	0.034 < 0.05	Ideal

Note: *df* = degrees of freedom, NFI = normed fit index, RFI = relative fit index, IFI = incremental fit index, TLI = Tucker–Lewis index, CFI = comparative fit index, PNFI = parsimony normed fit index, PCFI = parsimony comparative fit index, RMSEA = root mean square error of approximation.

**Table 6:** Results of model estimates.

Paths	Non-Standardised Path Coefficient	S.E.	C.R.	Standardised Path Coefficient
Value orientation→Environmental concern	0.488 **	0.227	2.148	0.216
Environmental concern→Consequence awareness	0.532 ***	0.134	3.977	0.314
Consequence awareness→Responsibility attribution	0.531 ***	0.199	2.671	0.284
Responsibility attribution→Individual norm	0.733 ***	0.279	2.633	0.448
Watershed belonging→Individual norm	0.128 ***	0.033	3.897	0.160
Social capital→Individual norm	0.448 ***	0.092	4.869	0.209
Watershed belonging→Watershed ecological protection behaviour	1.144 ***	0.104	11.023	0.872
Watershed belonging→Social capital	1.031 ***	0.241	4.286	0.694
Social capital→Watershed belonging	0.757 ***	0.279	2.712	0.469
Social capital→Watershed ecological protection behaviour	1.044 ***	0.104	10.026	0.788
Individual norm→Watershed ecological protection behaviour	1.203 ***	0.070	17.079	0.874
Value orientation→Watershed ecological protection behaviour	0.110	0.145	0.758	0.156
Environmental concern→Watershed ecological protection behaviour	0.036	0.024	1.531	0.019
Consequence awareness→Watershed ecological protection behaviour	0.090	0.099	0.914	0.045
Responsibility attribution→Watershed ecological protection behaviour	2.938	2.628	1.118	0.473

Note: S. E. = standard error, C. R. = critical ratio. ** and *** indicate significance at the 5% and 1% levels, respectively.

**Table 7:** Results of the mediating effect test.

Paths	Mediating Effect
Watershed belonging→Individual norm→Watershed ecological protection behaviour	0.140 ***
Social capital→Individual norm→Watershed ecological protection behaviour	0.183 ***

Note: *** indicates significance at the 1% level.

## Data Availability

All the data will be available from the corresponding author after reasonable request.
